# FUT11-Driven fucosylation coordinates K63 ubiquitination of keratin 17 to sustain psoriatic keratinocytes hyperproliferation

**DOI:** 10.1186/s12964-025-02422-6

**Published:** 2025-10-22

**Authors:** Xia Li, Guohao Li, Haijun Miao, Lixin Yue, Jixin Gao, Qingyang Li, Shuai Shao, Gang Wang, Erle Dang

**Affiliations:** Department of Dermatology, Xijing Hospital, Fourth Military Medical University, Xi’an, Shaanxi 710032 China

**Keywords:** Fucosylation, Fucosyltransferase 11 (FUT11), Psoriasis, Keratinocytes, Proliferation, Ubiquitination

## Abstract

**Background:**

The pathogenesis of psoriasis is characterized by dysregulated post-translational modifications, with particular emphasis on fucosylation—a glycosylation process mediated by fucosyltransferases (FUTs). Keratin 17 (K17), overexpressed in psoriatic keratinocytes, drives inflammation and proliferation, but its interplay with fucosylation remains unclear. This study aimed to elucidate the role of fucosylation in psoriasis, specifically focusing on the regulation of K17 stability by FUT11.

**Methods:**

To investigate fucosylation dynamics, we employed single-cell RNA sequencing (scRNA-seq) to analyze N-glycan biosynthesis activity in psoriatic versus healthy keratinocytes. Fucosylation levels were assessed in human and murine psoriatic lesions, as well as in cytokine-stimulated keratinocytes, using Aleuria aurantia lectin (AAL). An imiquimod (IMQ)-induced psoriasis-like mouse model and primary keratinocytes treated with psoriasis-associated cytokines (Pso-Mix) (IL-17, TNF-α, IL-1α, OSM and IL-22) were utilized to evaluate the effects of 2-fluorofucose (2-FF) and FUT11 siRNA. We further explored the mechanisms regulating K17 stability through immunoprecipitation, ubiquitination assays, and cycloheximide chase experiments.

**Results:**

Our findings revealed that psoriatic keratinocytes exhibited elevated levels of fucosylation, which correlated with upregulation of FUT11. Administration of 2-FF or silencing FUT11 significantly attenuated IMQ-induced inflammation, as evidenced by reductions in epidermal thickness, immune cell infiltration, and the expression of pro-inflammatory mediators such as IL-17A and CCL20. We demonstrated that FUT11 mediates α-1,3-fucosylation of K17, stabilizing it through K63-linked ubiquitination facilitated. Notably, silencing FUT11 disrupted the interaction between ubiquitination and fucosylation, leading to accelerated K17 degradation and a subsequent decrease in keratinocyte proliferation.

**Conclusions:**

Our results indicate that FUT11-driven fucosylation is integral to the stabilization of K17 via K63 ubiquitination, thereby perpetuating psoriatic inflammation. Targeting FUT11 or inhibiting fucosylation with 2-FF presents a novel therapeutic strategy for psoriasis management. This study highlights the critical interplay between glycosylation and ubiquitination in the pathophysiology of psoriasis, positioning FUT11 and K17 as pivotal targets for intervention.

**Graphical abstract:**

Our results indicate that FUT11-driven fucosylation is integral to the stabilization of K17 via K63 ubiquitination, thereby perpetuating psoriatic inflammation. Targeting FUT11 presents a novel therapeutic strategy for psoriasis management. This study highlights the critical interplay between glycosylation and ubiquitination in the pathophysiology of psoriasis, positioning FUT11 and K17 as pivotal targets for intervention.
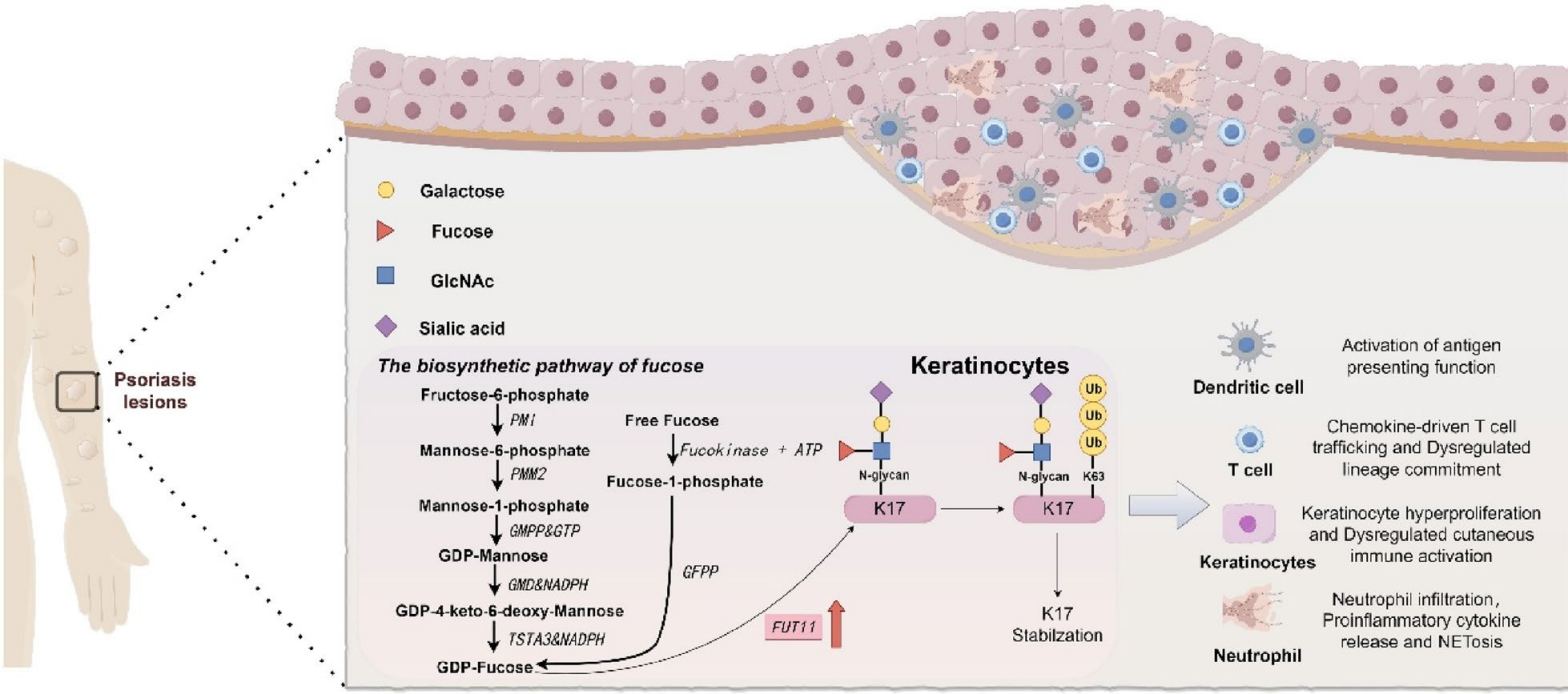

**Supplementary Information:**

The online version contains supplementary material available at 10.1186/s12964-025-02422-6.

## Highlights

1. FUT11 is the key fucosyltransferases that responsible for upregulated fucosylation in psoriatic keratinocytes.

2. FUT11-mediated fucosylation stabilizes K17 via K63-linked ubiquitination, driving keratinocyte hyperproliferation in psoriasis.

3. FUT11 orchestrates crosstalk between fucosylation and ubiquitination to sustain K17 overexpression.

4. Targeting FUT11 or fucosylation represents a novel therapeutic strategy for psoriasis.

**Supplementary Information:**

The online version contains supplementary material available at 10.1186/s12964-025-02422-6.

## Introduction

Protein glycosylation is a prevalent and essential posttranslational modification that plays a central role in various physiological and pathological processes, including chronic inflammatory disease and immune response [1–4]. Among the different forms of glycosylation, fucosylation stands out as a significant modification involving the conjugation of glycoproteins with sugar L-fucose (L-Fuc) at asparagine or serine/threonine residues (N- or O-linked, respectively). This process is mediated by a family of enzymes known as fucosyltransferases (FUTs), which modulate protein functions critical for immune and developmental processes [5]. Previous studies have established correlations between the abnormal growth of psoriatic epidermis and the presence of fucose-labeled glycoproteins derived from keratinocytes in psoriasis lesions [6]. However, the detailed mechanisms underlying the fucosylation alterations in psoriasis remain unclear. Keratin 17 (K17), a type I intermediate filament, is predominantly expressed in the basal cells of epithelia and has been identified as being specifically overexpressed in psoriatic keratinocytes [7, 8]. Our prior research demonstrated that elevated K17 enhances the biological activity of the keratinocytes, rendering them more proliferative and promoting the secretion of various chemokines (such as CXCL1, CCL20) through the PI3k/AKT/NF-κB signaling pathway. This process facilitates the recruitment of neutrophils to the lesion site, where they release a substantial amount of inflammatory cytokines, thereby initiate psoriatic inflammation [9]. Futhermore, activated keratinocytes expose K17 epitopes that activate T cells, which then migrate to the skin and secrete inflammatory cytokines such as IFN-γ, IL-22, and IL-17A [10]. These cytokines subsequently activate STAT1/3 and ERK1/2 pathways in keratinocytes, further upregulating K17 and exacerbating local inflammation, creating a vicious cycle that drives the progression of psoriasis [11]. Additionally, ubiquitination modification plays an important role in regulating K17 stability and mediating its overexpression in psoriasis. Specifically, the E3 ubiquitin ligase Trim21 in psoriatic skin lesions enhances stability of K17 through K63-linkage ubiquitination, thereby promoting the proliferation of keratinocytes [12]. However, whether K17 undergoes glycosylation modification and plays a role in the pathogenesis of psoriasis still remain unclear.

Fucosyltransferases are a family of enzymes that catalyzed the transfer of fucose from GDP-fucose to glycoconjugates [13]. Previous studies have shown that the FUT family is closely related to the onset and progression of inflammatory disease. Zhe Wang et al. found that FUT2 depletion in intestinal stem cells (ISCs) aggravated the epithelial damage and disrupted the growth and proliferation capacity of ISCs via escalating LPS-induced endoplasmic reticulum (ER) stress and initiating the IRE1/TRAF2/ASK1/JNK branch of unfolded protein response (UPR) [14]. Gerard Cantero-Recasens et al*.* found the expression of FUT8 is elevated in patients with ulcerative colitis (UC). FUT8 contributes to the pathogenesis of UC by modulating the biophysical properties of mucus through the regulation of MUC1 levels on the cell surface and the quantity and quality of secreted MUC2 and MUC5AC [15]. Additionally, FUT family is also closely related to the occurrence and development of psoriasis, such as FUT1 [16], FUT8 [17]. FUT1 deficiency has been shown to enhances imiquimod-induced psoriasis-like skin inflammation by promoting CXCL1 expression [16], whereas knockout of FUT8 has been found to ameliorate the phenotypes in IL-23 psoriasis-like mouse model [17]. These findings suggest that different members of the FUT family may have distinct roles in the pathophysiology of psoriasis, highlighting the complexity of their functions in inflammatory processes.

In this study, we report elevated fucosylation in psoriatic lesions. The application of the fucosylation inhibitor 2-FF significantly alleviated imiquimod-induced psoriasis-like phenotypes, including reduced erythema and scaling, and decreased epidermal thickness. Further investigation revealed that FUT11 mediated fucosylation of the psoriasis-specific K17 in particular, has an impact on keratinocytes hyperproliferation and pro-inflammatory cytokine production by modulating its ubiquitination and stablity. This novel finding reveals a hitherto unknown mechanism in amplification of inflammation centered on K17 and underscores the significant role of fucosylation in the pathology of psoriasis.

## Methods and materials

### Skin samples

We selected patients with psoriasis who presented at Xijing Hospital and had been free of systemic diseases or treatments for at least 6 weeks. All clinical tissue samples were collected with approval from the Ethics Committee of Air Force Medical University. Human specimen analyses strictly adhered to institutional guidelines and the Declaration of Helsinki. Written informed consent was obtained from all participants before skin lesion collection. Control biopsies consisted of healthy skin tissue discarded during cosmetic procedures from Donors in the Department of Plastic Surgery at Xijing Hospital. For psoriasis-like mouse models, skin and ear tissues were fixed in 4% paraformaldehyde at 4 °C overnight. Tissue sections were prepared from paraffin-embedded blocks at the pathology core facility.

### Experimental animals

Female C57BL/6 mice (6 weeks old) were obtained from the Experimental Animal Center of Air Force Medical University. Mice were housed in specific pathogen-free (SPF) facilities with controlled temperature (22 ± 1 °C), humidity (50 ± 5%), and 12-hour light/dark cycles, with ad libitum access to food and water. Animals were randomly assigned to experimental groups (*n* = 4 per group). All procedures were approved by the Institutional Animal Care and Use Committee (IACUC) and complied with the NIH Guide for the Care and Use of Laboratory Animals. The Dorsal skin of the mice was shaved at least 2 days before the beginning of treatments. The IMQ-induced psoriasis model was established as previously described. Briefly, 62.5 mg of 5% imiquimod (IMQ) cream (Aldara^®^; iNova Pharmaceuticals, Sydney, Australia) was applied daily to shaved Dorsal skin for 5 consecutive days. Controls received an equivalent amount of petroleum jelly (Vaseline^®^; Unilever, London, UK).

To investigate the role of fucosylation in psoriasis, we used a cream-based mixture of the fucosylation inhibitor 2-FF (8 mg/kg/d, #sc-36700, SynChem-Formosa, Elk Grove Village, USA) or an equal volume DMSO with emulsion base in shaved mouse backs in the morning and treated with 62.5 mg of 5% IMQ cream or Vaseline in the afternoon, for a duration of 5 days.

To establish the IMQ-induced psoriasis model in the mouse ears, the right ear received a topical Dose of 20mg IMQ (Aldara^®^; iNova Pharmaceuticals, Sydney, Australia) for 5 consecutive days, whereas the control mice received the same dose of vehicle (Vaseline^®^; Unilever, London, UK). To investigate the role of fucosyltransferase FUT11 in the pathogenesis of psoriasis, we used the siRNA targeting FUT11 (2.5 nmol/kg/d, Ribobio Biotechnology, Guangzhou, China) were individually mixed with vehicle and applied topically to the right ear of mice after 3–4 h of challenge with IMQ every other day for 5 days. Control group mice were treated with negative control siRNA (2.5 nmol/kg/d, Ribobio Biotechnology, Guangzhou, China). Sequences of the siRNAs are listed in Supplementary Table S2.

### scRNA-Seq and data analysis

Publicly available scRNA-seq datasets from the study Developmental cell programs are co-opted in inflammatory skin disease were utilized (accession: https://developmentcellatlas.ncl.ac.uk/datasets/hca_skin_portal*)*, encompassing skin specimens from healthy adults and psoriasis patients. A random subset of 50,000 cells was selected from pre-processed data for downstream analysis.

All computational analyses were performed in R (v4.4.0). Dimensionality reduction visualization using Uniform Manifold Approximation and Projection (UMAP) embeddings were generated using the CellDimPlot function from the SCP package (v0.5.6). Gene expression profiling was performed by visualizing feature patterns with scCustomize’s FeaturePlot_scCustom function (v3.0.1). N-glycan biosynthesis pathway activity scores were computed using the AddModuleScore function form the Seurat package (v5.1.0) based on scaled mean expression values of 39 core genes defined in the glycosylation mapping resource Global Mapping of Glycosylation Pathways in Human-Derived Cells. Group differences in pathway activity scores were evaluated using Kruskal-Wallis non-parametric testing, with visualization implemented by ggboxplot from ggpubr (v0.6.0).

### Cell culture

This study has obtained approval from the Ethics Committee of Air Force Medical University. Written informed consent has been obtained from all donors. The human keratinocyte cell line HaCaT, purchased from KeyGEN Biotech (GB300, Nanjing, China), was cultured in a humidified incubator at 37 °C with 5% CO₂, and supplemented with 10% fetal bovine serum (FBS) to support its growth and proliferation. After a 24-hour starvation period, cells at a confluence of 40–60% were stimulated with cytokines, including IL-17, IL-22, tumor necrosis factor-α (TNF-α), IL-1α, and oncostatin M (OSM), at concentrations of 50, 20, 50, 20, and 20 ng/mL, respectively (Sino Biological Inc, Beijing, China). The cells were incubated with these cytokines for 48 h, while cells treated with PBS served as the negative control.

Human primary KCs were collected from the foreskins of twelve patients (aged 8 to 30 years) who underwent urological surgery at the Department of Urology, Xijing Hospital, as previously [18]. Keratinocyte complete media (EpiLife, Thermo Fisher Scientific, USA) supplemented with EpiLife medium + 60 µM calcium/EpiLife Defined Growth Supplement (EDGS, Thermo Fisher Scientific, USA) was used to sustain human primary KCs. Penicillin‒streptomycin (50 U/mL) was added. Human primary KCs were then treated with FUT11 siRNA, 1 mM of 2-FF inhibitor (#sc-36700, SynChem-Formosa, Elk Grove Village, USA) in 6-well plates.

### Small interfering RNA

siRNA oligos against human FUT11 were purchased from GenePharma (Shanghai, China). Sequences of the siRNAs are listed in Supplementary Table S2.

### Real-time quantitative PCR

The isolation of total RNA from cell or tissue samples was carried out using the TRIzol Reagent (Thermo Fisher Scientific, Waltham, USA), adhering strictly to the manufacturer’s guidelines. Reverse transcription of the RNA samples into cDNA was achieved using the PrimeScript™ RT Reagent Kit from Takara (Otsu, Japan). Real-time quantitative PCR (RT-qPCR) was performed using the SYBR Green PCR Master Mix from Takara (Otsu, Japan) on a CFX384 real-time quantitative PCR detection system from Bio-Rad (Hercules, CA). The specific primers used in this study are listed in Supplementary Table S1. Each reaction was replicated three times across at least three independent experiments. The relative gene expression levels were normalized against human β-Actin or mouse β-Actin and calculated using the comparative Ct (2⁻∆∆CT) method.

### Western blot analysis

Cell lysates were prepared using RIPA lysis buffer (Runde Biologicals Ltd, China) containing 1 mM phenylmethylsulfonyl fluoride and a phosphatase inhibitor. Nuclear and cytoplasmic proteins were isolated using a nuclear and cytoplasmic protein extraction kit (#P0028, Beyotime Biotechnology, Shanghai, China). Protein concentration was determined using a Pierce BCA Protein Assay Kit (Pierce Biotechnology, Waltham, USA). Subsequently, 15–20 µg of cell lysate samples were separated by 10–15% SDS-PAGE and transferred onto PVDF membranes (Millipore, Burlington, USA). Membranes were blocked with blocking buffer for 1 h and then incubated with the specified primary antibodies overnight at 4 °C: anti-K17 (1:1000, ab53707, Abcam, Cambridge, UK), Biotin-Aleuria Aurantia Lectin (AAL) (1:2000, B-1395, Vector Laboratories, Newark, USA), anti-FUT11(1:100, ab121411, Abcam, Cambridge, UK), anti-CyclinD1(1:1000, 2922, Cell Signaling Technology, Boston, USA), anti-GAPDH (1:5000, 5174, Cell Signaling Technology, Boston, USA), anti-β-Tubulin (1:5000, 2146, Cell Signaling Technology, Boston, USA) and anti-Trim21(1:1000, 67136-1-Ig, Proteintech, Chicago, USA). After washing the membranes, they were incubated with HRP-conjugated secondary antibodies for 1 h at room temperature. Protein expression levels were measured using Image Lab software version 5.2.1 (Bio-Rad Laboratories, Inc.).

### Co-immunoprecipitation

Cell pellets were gathered and lysed subsequently in a lysis buffer containing Halt Protease and Phosphatase Inhibitor Cocktail from Thermo Fisher Scientific (Waltham, USA). The cell lysates were incubated with anti-K17 (#sc-393002, Santa Cruz, Dallas, USA) for 3 h at 4 °C, followed by incubation with Protein A/G PLUS-Agarose (#sc-2003, Santa Cruz, Dallas, USA) on a rocker platform at 4 °C overnight. The beads were washed four times with cold PBS. To conduct an immunoblot analysis, the supernatant was aspirated and discarded, and the pellet was resuspended in 1x electrophoresis sample buffer.

### Hematoxylin and Eosin (H&E) staining

The tissue samples from humans and mice were fixed, paraffin-embedded, and cut into 8 μm sections, which were then dewaxed using EZ-DeWax™ (BioGenex, Fremont, USA). The sections were then stained with hematoxylin and eosin (H&E) for histological studies. After scanning with a slide scanner (Hamamatsu Photonics, Shizuoka, Japan), the slides were studied using NDP2 viewer software (Hamamatsu Photonics, Shizuoka, Japan).

### EdU proliferation assay

HaCaT cells were seeded in quadruplicate in a 96-well plate at a density of 5 × 10³ cells per well. Following 24 h of incubation, the cells were transfected with siRNA, plasmid, or a combination of ENOBlock and BI-D1870 for 48 h. Subsequently, an EdU cell proliferation assay kit (RiboBio, Guangzhou, China) was utilized to assess cell proliferation. A fluorescence microscope (Olympus, Tokyo, Japan) was employed to determine the percentage of EdU-incorporated cells. The EdU-positive cells in each group were counted by selecting five random fields of view.

### Immunofluorescence and immunohistochemical staining

Biopsies obtained from lesional skin of psoriasis patients and healthy control skin or IMQ-induced mice were fixed in 4% paraformaldehyde and embedded in paraffin. For immunofluorescence staining, cells or skin biopsy specimens were incubated overnight at 4 °C with the following primary antibodies: anti-K17 (1:1000, ab53707, Abcam, Cambridge, UK), Biotin-Aleuria Aurantia Lectin (AAL) (1:500, B-1395, Vector Laboratories, Newark, USA). After washing three times with PBS, the cells were incubated with FITC-conjugated secondary antibodies and streptavidin CY3(1:5000, SA-1300, Vector Laboratories, Newark, USA). After Hoechst (1:1000, Solarbio Technology, Beijing, China) was applied to all cells to label the nuclei. Samples were analyzed using a confocal microscope (LSM880, Carl Zeiss, Germany). For immunohistochemical staining, tissue sections were incubated with 0.3% H₂O₂ for 10–20 min and then incubated overnight at 4 °C with one of the following primary antibodies: anti-K17 (1:1000, ab53707, Abcam, Cambridge, UK), Biotin-Aleuria Aurantia Lectin (AAL) (1:500, B-1395, Vector Laboratories, Newark, USA), Biotin-Ulex Europaeus Agglutinin I (UEA I) (1:500, B-1065, Vector Laboratories, Newark, USA), anti-FUT11(1:100, ab121411, Abcam, Cambridge, UK), anti-Ki67 (1:1000, ab15580, Abcam, Cambridge, UK), anti-CD3 (1:200, ab16669, Abcam, Cambridge, UK) and anti-Ly6G (1:50, sc-53515, Santa Cruz Biotechnology, Dallas, USA). Subsequently, the sections were incubated with HRP-labeled goat anti-mouse/rabbit (1:200, CWBIO, Beijing, China) at room temperature for 1 h. DAB (Gene Tech, Shanghai, China) was used to detect biotinylated antibodies.

### Flow cytometry analysis

Cells ​​were harvested​​ by trypsinization and ​​centrifuged​​ at 300 g for 5 min at 4 °C. ​​After two washes​​ with PBS, cells ​​were resuspended​​ at a density of 1–5 × 10⁶ cells/mL in PBS. ​​A 100-µL aliquot​​ of the cell suspension ​​was transferred​​ to flow tubes, and biotin-conjugated primary antibody AAL (1:100, B-1395, Vector Laboratories, Newark, USA) ​​was added​​. ​​The mixture was incubated​​ at 4 °C in the dark for 20 min. ​​Cells were then washed​​ twice with PBS containing 2% FBS, followed by the addition of streptavidin-CY3 (1:100, SA-1300, Vector Laboratories, Newark, USA) and ​​incubation​​ for another 20 min in the dark. ​​After two additional washes​​, cells ​​were resuspended​​ in 300 µL PBS and ​​filtered​​ through a 40-µm nylon mesh to remove clumps. Data ​​were acquired​​ on a flow cytometer. ​​Viable cells were gated​​ based on forward/side scatter profiles, and CY3 fluorescence intensity ​​was analyzed​​ to determine antigen expression. ​​The percentage of positive cells was calculated​​ using FlowJo software.

### PNGaseF enzymatic deglycosylation of glycoproteins

Total cellular proteins were quantified by BCA assay. For each reaction, 20 µg of protein sample was denatured in 4 µL of 1× denaturation buffer (50 mM Tris-HCl, pH 7.5, 1% SDS, 10% glycerol) by heating at 95 °C for 5 min, then cooled on ice. To neutralize SDS inhibition, 1 µL of NP-40 (final concentration 0.5%) and 2 U of PNGaseF (#P0704, New England Biolabs, Ipswich, USA) were added, along with 5 µL of 10× Glycoprotein Denaturing Buffer (supplied with the enzyme), bringing the total volume to 50 µL. Reactions were incubated at 37 °C for 24 h with gentle shaking. Negative controls omitted PNGaseF to assess background. Reactions were terminated by adding 5× SDS-PAGE loading buffer and heating at 95 °C for 5 min, followed by analysis via Western blot or mass spectrometry to evaluate glycan removal by molecular weight shift or peptide mass fingerprinting.

### Protein stability assay

For the protein stability assay, HaCaT cells were treated with 10 µM cycloheximide (CHX, Cell Signaling Technology, Boston, USA) for 0, 2, 4, 6, 8, and 10 h before harvesting.

### Statistical analyses

For statistical analysis, data obtained from at least three independent experiments were processed using GraphPad Prism software version 8 (GraphPad Software, San Diego, USA). Statistical significance (*P* < 0.05, *P* < 0.01, *P* < 0.001, *P* < 0.0001) was determined using Student’s unpaired two-tailed t-test or Analysis of Variance (ANOVA) as indicated in the legends. Flow cytometry data were analyzed using FlowJo v10. The sample size, denoted as “n,” is indicated in the legends.

## Results

### Abnormal fucosylation pattern occurred in psoriatic keratinocytes

**Fig. 1:**
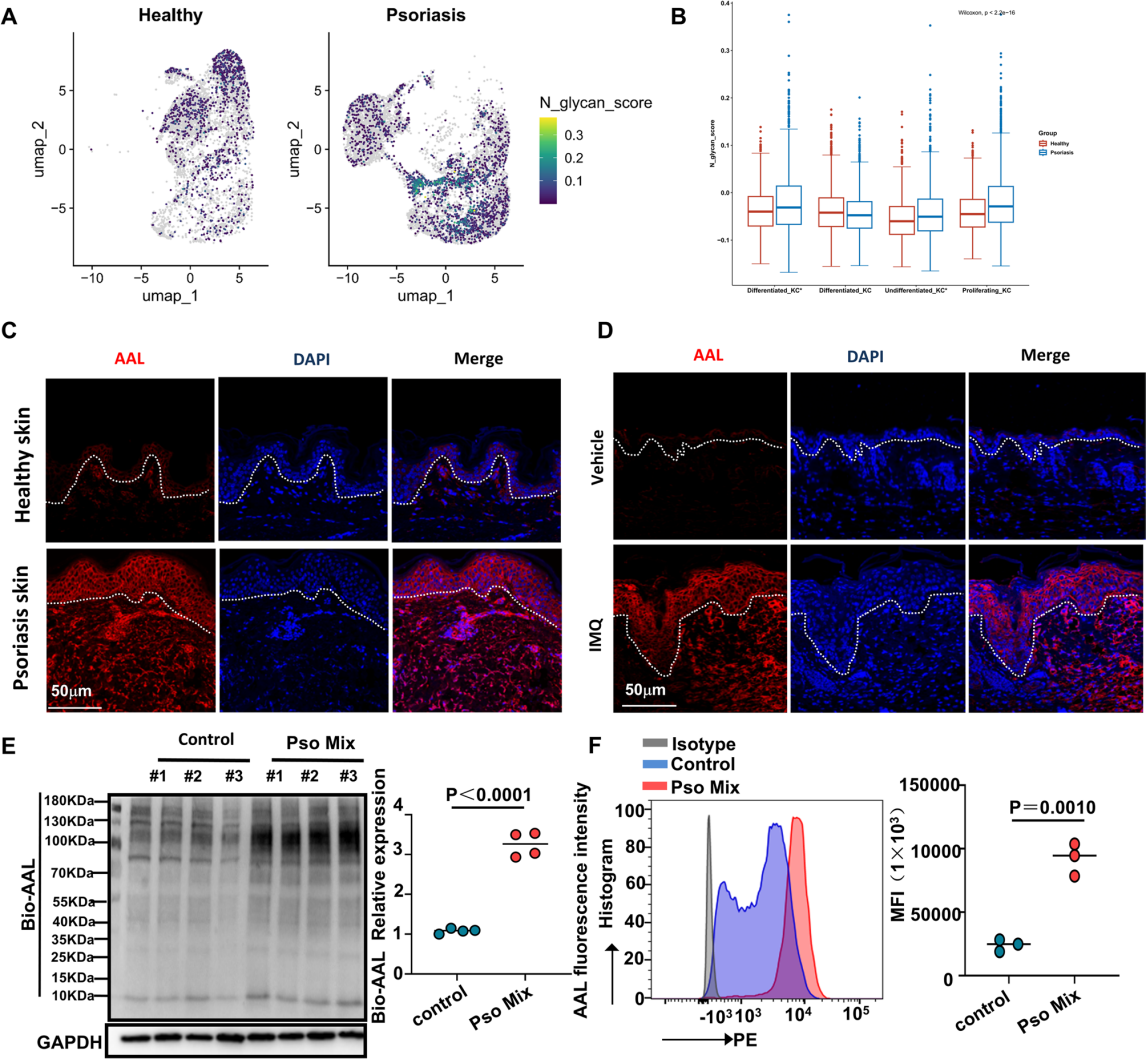
The occurrence of fucosylation modification in psoriatic keratinocytes (KCs). **A** Single-cell N-glycan biosynthesis activity was scored in keratinocyte subpopulations using the AddModuleScore function in Seurat. UMAP plots visualize cell score distribution with color gradients reflecting scoring intensity. **B** Box-and-whisker plots visualize the N-glycan biosynthesis activity scores within each keratinocyte subsets (Undifferentiated KC*, Proliferating KC, Differentiated KC, and Differentiated KC*), grouped by health and psoriasis. **C** Immunofluorescence staining of AAL (red) in normal and psoriatic skin. Scale bars = 50 μm (*N* = 10 patients). **D** Epidermal AAL (red) is expressed in IMQ mouse lesional skin compared with WT skin, scale bar = 50 μm (*N* = 10 mice). **E** Lectin blot analysis of fucosylation in primary KCs stimulated by the Pso-Mix. **F** The mean fluorescence intensity (MFI) of AAL in peripheral keratinocytes from control group (*n* = 3) and Pso-Mix treated group (*n* = 3). Results are shown as mean ± SD. *P < 0.05, **P < 0.01, ***P < 0.001

To investigate the potential role of abnormal N-glycosylation in the development of psoriasis, we analyzed scRNA-seq data sets obtained from 5 healthy Donors and 3 psoriatic patients [19] (Figure S1A). By reviewing the literature [20], we constructed a core gene set of 39 genes involved in N-glycan synthesis. Based on this core gene set, we employed the AddModuleScore function to score the N-Glycan-Biosynthesis activity across all individual cells. The results showed that the N-glycan biosynthesis pathway was significantly active in the KC subpopulation of psoriasis (Figure S1B). Subsequently, we specifically evaluated the N-Glycan-Biosynthesis activity within the keratinocyte subpopulation. The analysis revealed notably heightened activity of this pathway in the proliferative cell subpopulation of psoriasis (Fig. 1A and B). As an important subtype of N-glycosylation, fucosylation has been previously implicated in the abnormal growth of the psoriatic epidermis, potentially associated with fucosylated glycoproteins in keratinocytes of psoriatic lesions [6]. To further explore fucosylation changes in psoriasis, we utilized aleuria aurantia lectin (AAL), a carbohydrate probe for detecting fucosylation in glycoproteins. Immunofluorescence analysis demonstrated significantly elevated fucosylation levels in the lesional skin of psoriatic patients (Fig. 1C) and in the imiquimod (IMQ)-induced psoriasis-like mouse model (Fig. 1D). In contrast, glycosylation patterns recognized by UEA-I did not show significantly changes (Fig. S1 and D). Notably, AAL recognizes fucosyl residues linked via α(1,2), α(1,3) and α(1,6) bonds, while UEA-preferentially binds to oligosaccharides containing α(1,2) fucose [21]. Additionally, AAL blotting revealed increased fucosylation in primary KC cells stimulated with psoriasis-associated cytokines (Pso-Mix) (IL-17, TNF-α, IL-1α, OSM and IL-22) (Fig. 1E). Meanwhile, we observed increased surface expression of fucosylation levels recognized by AAL on Pso-Mix keratinocytes compared with controls as measured by mean fluorescence intensity (MFI) of AAL. These results suggest an abnormal fucosylation pattern occurs in keratinocytes from psoriatic lesions, highlighting its potential role in the pathophysiology of psoriasis.

### Inhibition of fucosylation mitigates IMQ-induced psoriasis-like mice skin inflammation

**Fig. 2:**
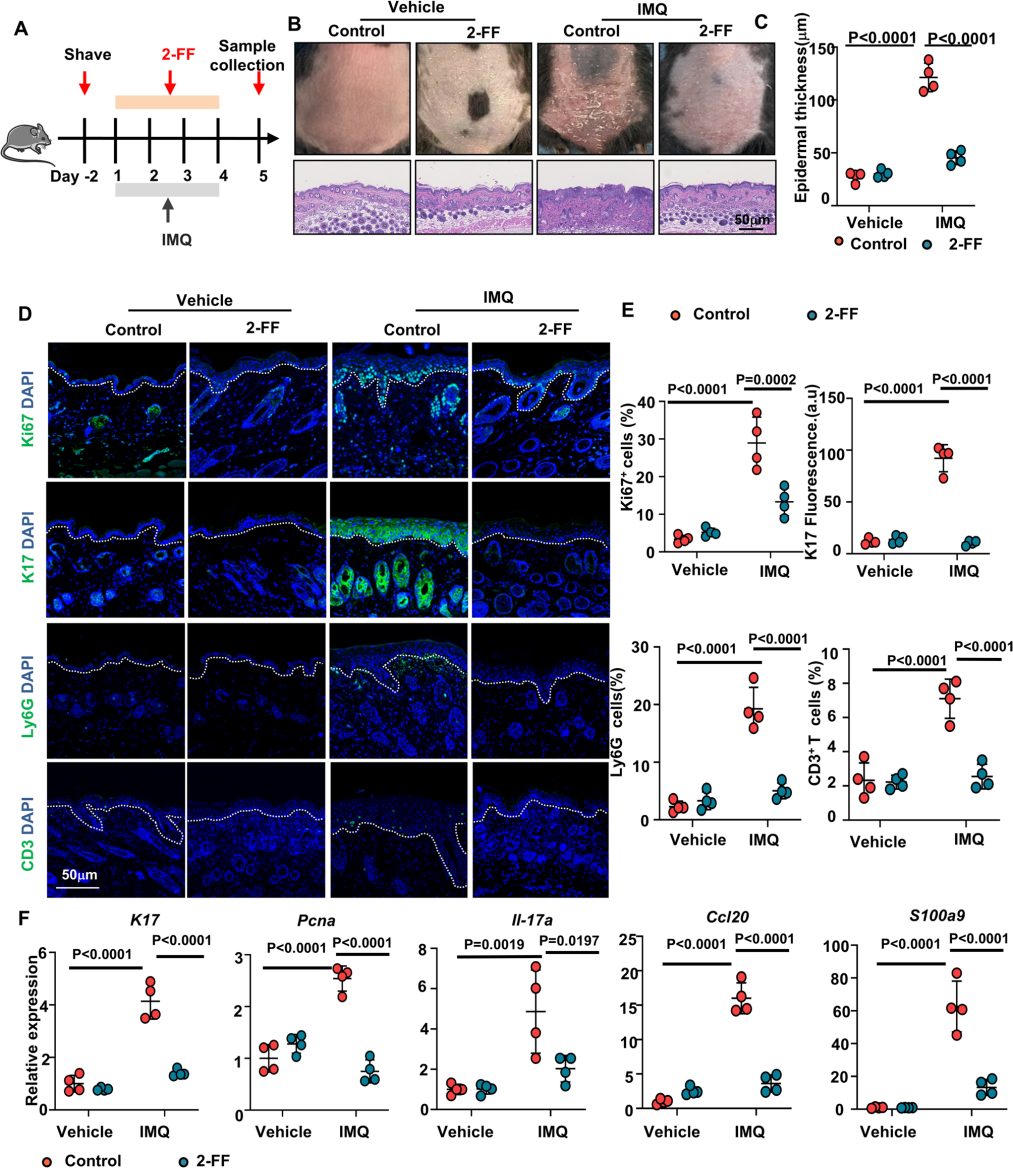
Inhibition of fucosylation significantly alleviates skin inflammation in IMQ-induced psoriasis-like mice. **A** Schematic diagram of the mouse experimental protocol. Mice in the IMQ group were topically treated with a fucosylation inhibitor 2-FF in the morning and IMQ in the afternoon, with *n* = 4 mice per group. **B** Phenotypes and representative H&E staining of IMQ-treated mice in the specified groups on day 5. The control group received topical application of Vaseline cream. Scale bars = 50 μm. *n* = 4 mice. **C** Epidermal thickness. **D** Immunofluorescence staining for Ki67, K17, Ly6G, and CD3, and **E** Quantification of Ki67+, Ly6G+, and CD3 + cells, as well as mean fluorescence intensity of K17, with *n* = 4 mice. Ki67 (green), KRT17 (green), Ly6G (green), CD3 (green), and nuclei stained with DAPI. Scale bars = 50 μm. **F** Relative mRNA expression of* K17, Pcna, Il-17a, Ccl20,* and *S100a9*. Results are shown as mean ± SD. *P < 0.05, **P < 0.01, ***P < 0.001

To evaluate the contribution of fucosylation increasing to psoriasis inflammation, we employed fucosylation inhibitor on the acute imiquimod (IMQ)-induced inflammatory skin model (Fig. 2A). Local application of fucosylation inhibitor 2-FF ameliorated psoriasis-like lesions in IMQ-treated mice, leading to reduced erythema, scaling, and epidermal thickness (Fig. 2B and C). Additionally, immunofluorescence results indicated a decrease in Ki67 and K17 expression following local application of fucosylation inhibitor 2-FF. Concurrently, the infiltration of Ly6G^+^ neutrophils and CD3^+^ T cells was decreased (Fig. 2D and E), consistent with quantitation analysis based on H&E staining. Additionally, mRNA expression of prolifertaion makers, pro-inflammatory mediators and antibacterial peptide in skin lesions, including *K17*,* Pcna*,* Il-17a*,* Ccl20* and *S100a9*, was suppressed by fucosylation inhibition (Fig. 2F).

### Fucose maintains the proliferation capacity of keratinocytes

**Fig. 3:**
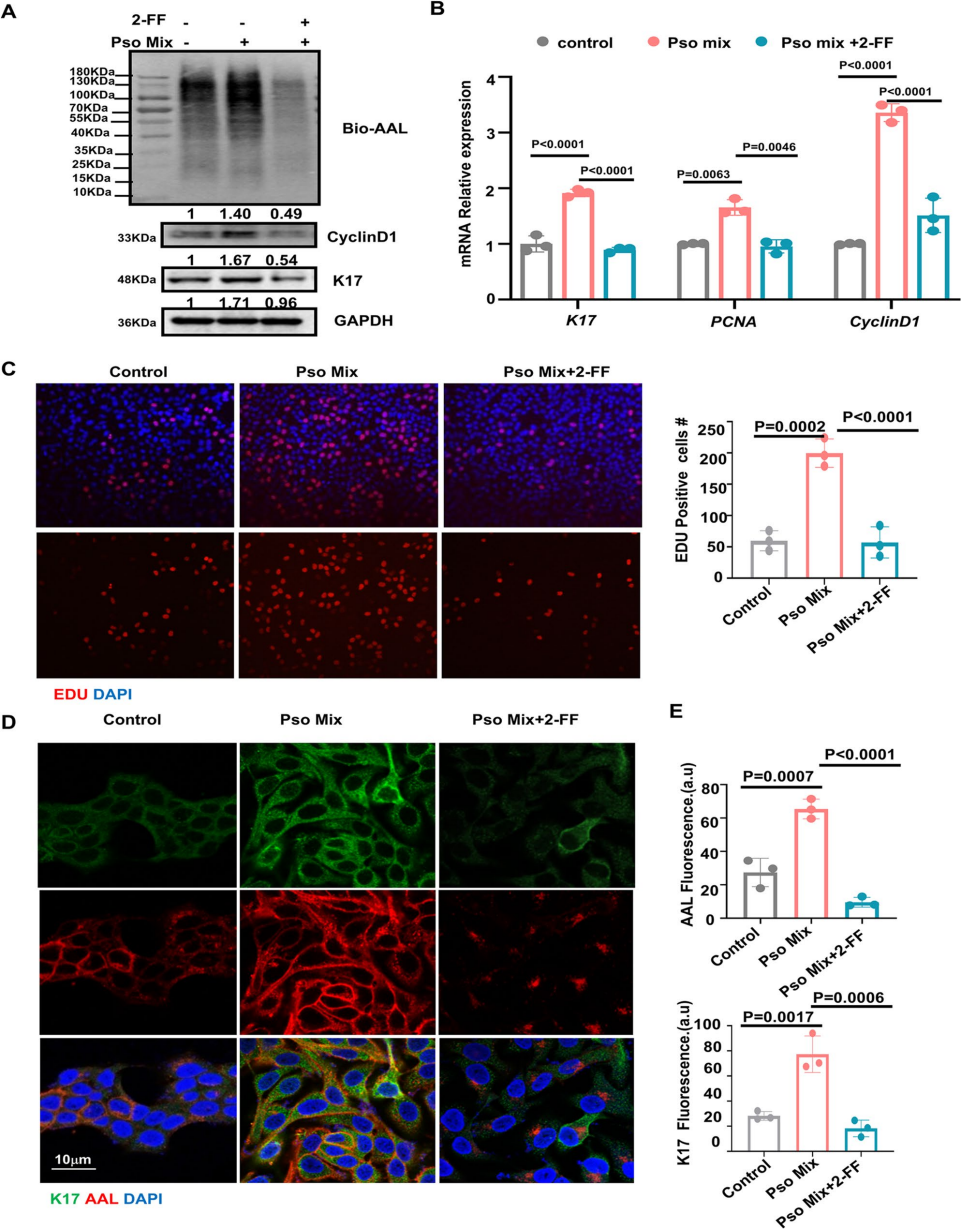
Fucose maintains the proliferation capacity of keratinocytes. **A** Lectin blot analysis and Western blot analysis of proteins related to cell proliferation, including K17 and CyclinD1 in KC cells. **B** Relative mRNA expression of *K17*, *PCNA*, and *CYCLIN* *D1*. **C** EdU assay and the percentage of EdU-positive cells. At least 3 independent experiments were conducted. **D** Immunofluorescence staining for AAL and K17. AAL (Red), K17 (green). Scale bars = 50 μm. **E** The mean fluorescent intensity of AAL and K17 was analyzed in the statistical chart. Results are shown as mean ± SD. *P < 0.05, **P < 0.01, ***P < 0.001

To explore how fucose modulates the function of KCs, we stimulated primary keratinocytes with Pso-Mix to mimic the psoriatic profile. Our previous work established that K17 can regulate keratinocytes proliferation in psoriasis. Pso-Mix treated keratinocytes exhibited a sustained K17 expression, whereas treatment with the fucosylation inhibitor significantly restored K17 levels during Pso-Mix stimulation (Fig. 3A). AAL blotting indicated a decrease in core fucosylation in keratinocytes stimulated with fucosylation inhibitor. Furthermore, the expression of *CYCLIN D1* also mirrored this trend. The mRNA levels of stemness-related genes including *K17*,* PCNA* and *CYCLIN D1* were restored by fucosylation inhibitor in Pso-Mix treatd keratinocytes (Fig. 3B). The fucosylation inhibitor 2-FF-treated keratinocytes exhibited diminished EdU signaling intensity, reflecting reduced proliferation capacity compared to the Pso-Mix treated group (Fig. 3C). Furthermore, immunofluorescence analyses confirmed that K17 expression was upregulated in treatment with Pso-Mix treated keratinocytes and Downregulated in those treated with 2-FF, alongside AAL blotting results indicating fucosylation levels (Fig. 3D and E). Collectively, these results concurrently revealed that fucose is crucial for maintaining the proliferation capacity of keratinocytes in the context of Pso-Mix-induced psoriasis-like injury.

### FUT11 is required for keratinocytes proliferation in psoriasis

**Fig. 4:**
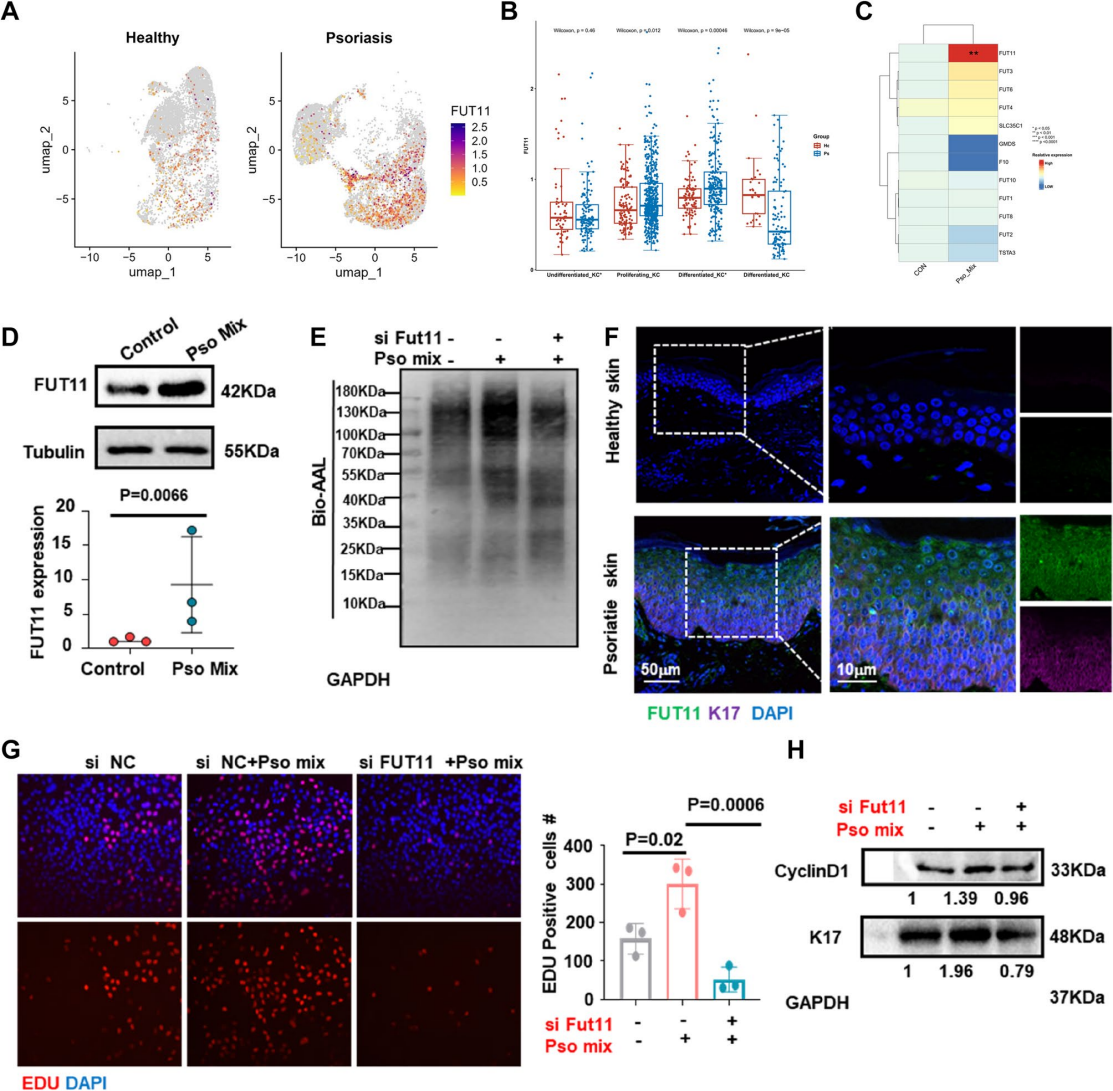
FUT11 is required for keratinocytes proliferation in psoriasis. **A** UMAP plot visualizes the expression levels of fucosyltransferase FUT11 across keratinocyte subpopulations. Gene expression intensity is represented through a color gradient, where darker colors correspond to higher expression levels. **B** Box-and-whisker plots visualize the distribution of FUT11 expression levels within each keratinocyte subset (Undifferentiated KC*, Proliferating KC, Differentiated KC, and Differentiated KC*), grouped by health and psoriasis. **C** Relative mRNA expression of *FUT1*, *FUT2*, *FUT3*, *FUT4*, *FUT6*,* FUT8*, *FUT10*, *FUT11*, *GMDS*, *TSTA3*, *SLC35C1* and *F10*. **D** Western blot analysis of protein FUT11. **E** Lectin blot analysis of bio-AAL. **F** Immunofluorescence staining for FUT11and K17. FUT11 (green), K17(purple). Scale bars = 50 μm, 10 μm. **G** EdU assay and the percentage of EdU-positive cells. **H** Western blot analysis of proteins related to cell proliferation, including K17 and CyclinD1 in KC cells. At least 3 independent experiments were conducted. Results are shown as mean ± SD. *P < 0.05, **P < 0.01, ***P < 0.001

To explore the precise mechanism underlying the elevated fucosylation in psoriatic lesions, we screened the expression of fucosylation-related glycosyltransferases and genes involved in fucose synthesis. scRNA sequencing analysis revealed that FUT11 was significantly upregulated in psoriasis keratinocytes subgroups (Fig. 4A), whereas other fucosylation-related glycosyltransferases did not exhibit clear difference (Figure S2). Further investigation revealed that FUT11 expression was significantly enriched in proliferating keratinocyte subpopulations (Fig. 4B), suggesting its potential role in regulating the abnormal fucosylation and hyper-proliferation of psoriasis keratinocytes. Consistently, both the protein and mRNA levels of *FUT11* were elevated in keratinocytes following Pso-Mix treatment (Fig. 4C and D). AAL blotting confirmed that silencing FUT11 via siRNA significantly reduced Pso-Mix induced core fucosylation in keratinocytes (Fig. 4E). Immunofluorescence analysis further confirmed a marked increase in FUT11 expression in psoriatic lesions compared to healthy controls, with FUT11 expression showing colocalization with the proliferation marker K17 (Fig. 4F). We further investigated the role of FUT11 in the proliferation of keratinocytes. EdU assays showed that silencing FUT11 suppressed the proliferation of keratinocytes induced by Pso-Mix (Fig. 4G). Moreover, levels of proliferation marker cyclin D1 and K17 were significantly decreased in KCs with FUT11 silencing (Fig. 4H). Collectively, these findings suggest that FUT11 plays a critical role in mediating both fucosylation and the proliferation of keratinocytes in the context of psoriasis.

### Silencing FUT11 allevate IMQ-induced psoriasis-like mice skin inflammation

**Fig. 5:**
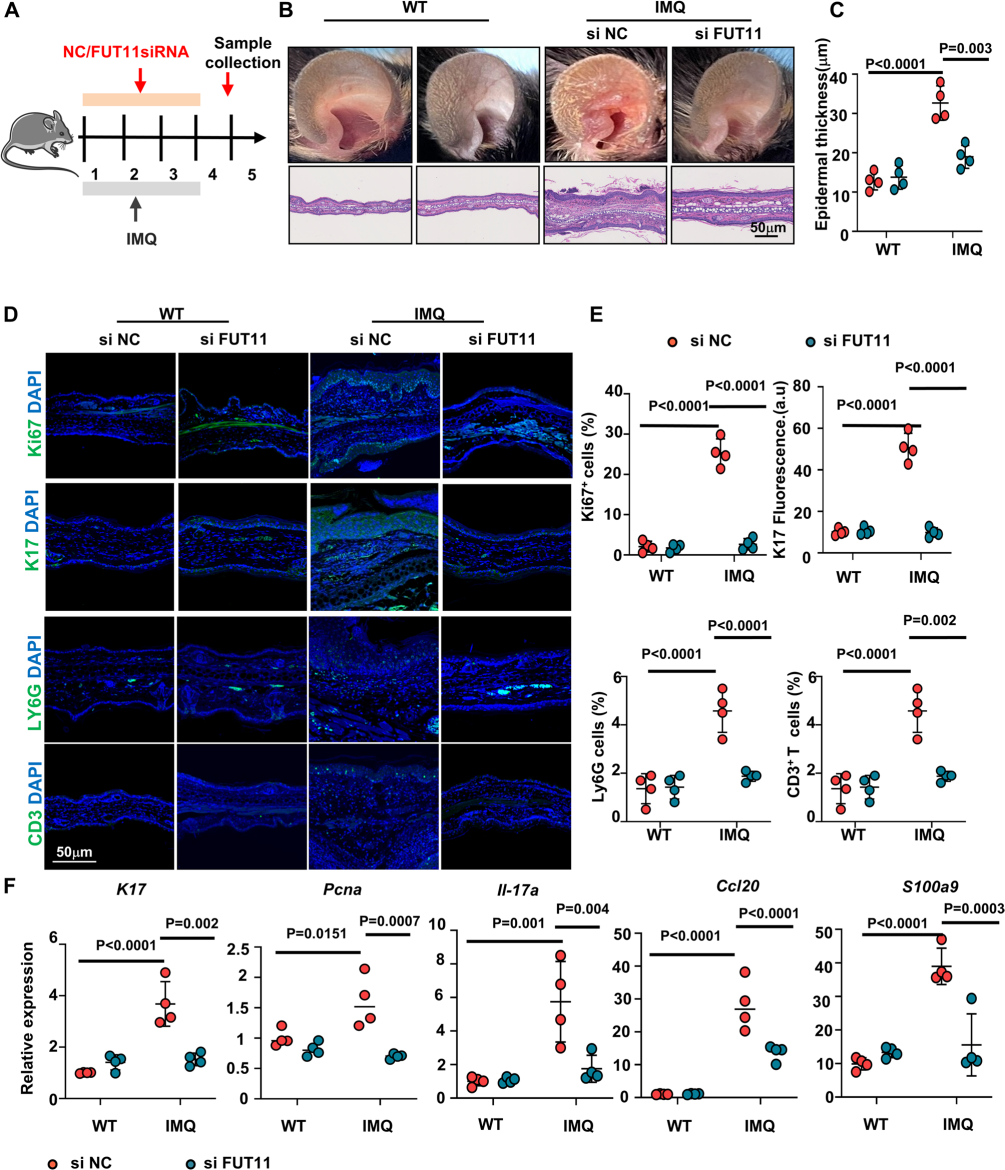
Inhibition of FUT11 mitigated skin inflammation in IMQ-induced psoriasis-like mice. **A** Schematic diagram of the mouse experimental protocol. Mice in the IMQ group were topically treated with FUT11 siRNA in the morning and IMQ in the afternoon, with *n* = 4 mice per group. **B** Phenotypes and representative H&E staining of IMQ-treated mice in the specified groups on day 5. The control group received topical application of Vaseline cream. Scale bar = 50 μm. *n* = 4 mice. **C** Epidermal thickness. **D** Immunofluorescence staining for Ki67, K17, Ly6G, and CD3. **E** Quantification of Ki67+, Ly6G+, and CD3 + cells, as well as mean fluorescence intensity of K17, with *n* = 4 mice. Ki67 (green), K17 (green), Ly6G (green), CD3 (green), and nuclei (DAPI). Scale bars = 50 μm. **F** Relative mRNA expression of *K17*, *Pcna*, *Il-17a*, *Ccl20*, and *S100a9*. Results are shown as mean ± SD. *P < 0.05, **P < 0.01, ***P < 0.001

To assess the role of FUT11 in psoriatic inflammation, we obseved the skin inflammation after silencing FUT11 in IMQ-induced psoriasis-like mice model (Fig. 5A). Relative mRNA expression of FUT11 was successfully suppressed in the ears of IMQ-treated mice following topical application of FUT11 siRNA (Figure S3). Notably, local application of FUT11 siRNA ameliorated psoriasis-like lesions in IMQ-treated mice, evidenced by relieved the symptoms of erythema, scaling, and epidermis thickness (Fig. 5B and C). Additionally, immunofluorescence results showed decreased expression of Ki67 and K17, along with increased K1 expression, following local application of FUT11 siRNA. Concurrently, the infiltration of Ly6G^+^ neutrophils and CD3^+^ T cells was significantly decreased in FUT11 siRNA transfected IMQ mice (Fig. 5D and E). Similarly, mRNA expression of prolifertaion makers, pro-inflammatory mediators and antibacterial peptide in skin lesions, including *K17*,* Pcna*,* Il-17a*,* Ccl20* and *S100a9* was suppressed by FUT11 inhibition (Fig. 5F).

### FUT11 affected K17 stability of by modulating fucosylation and ubiquitination

**Fig. 6:**
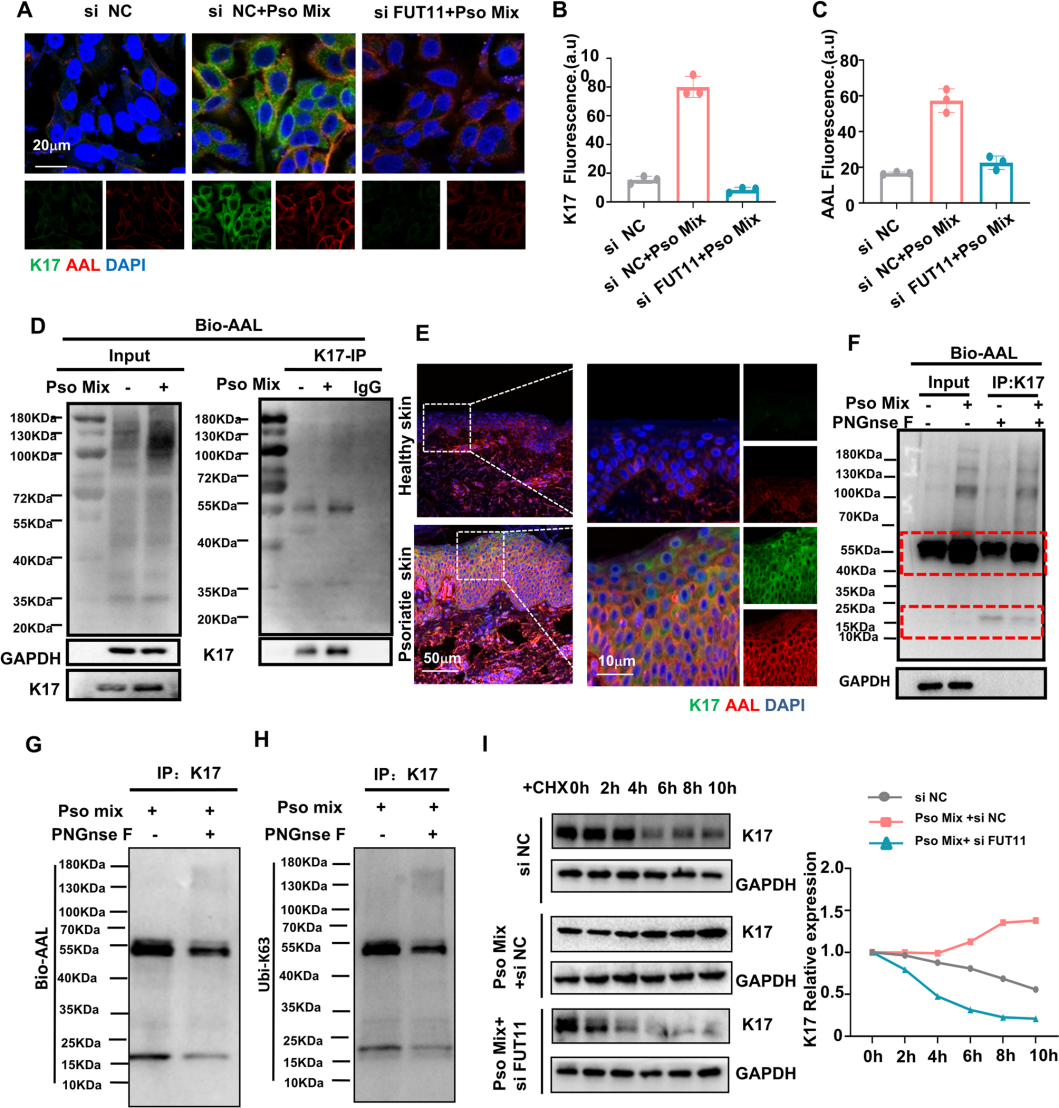
FUT11 affects the stability of K17. **A** Immunofluorescence staining for AAL and K17. AAL (red), K17 (green). Scale bars = 20 μm. **B** and **C** The mean fluorescent intensity of AAL and K17 was analyzed in the statistical chart. **D** Fucosylation modification of K17. Cells were immunoprecipitated with anti-K17 and immunoblotted as shown. **E** Immunofluorescence staining of AAL (red) in normal and psoriatic skin. Scale bars = 50 μm, 10 μm (*N* = 10 patients). **F** Western blot analysis of K17 digestion by PNGaseF enzyme. **G** Immunoprecipitation with anti-K17 antibody for bio-AAL. **H** Immunoprecipitation with anti-K17 antibody for Ubiquitination at K63 site. **I** KCs were treated with FUT11 siRNA under Pso-Mix condition for 24 h before treating with 10 µM cycloheximide (CHX). Whole-cell lysates were subjected to IB analysis. Results are shown as mean ± SD. *P < 0.05, **P < 0.01, ***P < 0.001

Considering the important role of keratin 17 in psoriasis and the co-localization of K17 and AAL labeled recognized in keratinocytes, we speculate that FUT11 may participate in psoriasis by regulating K17 fucosylation. Immunofluorescence analysis demonstrated that inhibition of FUT11 led to a significant downregulation of K17 expression and a decrease in fucosylation levels (Fig. 6A, B and C). We further employed immunoprecipitation to examine whether K17 undergoes fucosylation modification. Our results confirmed that K17 is subject to AAL-recognized fucosylation modification, with levels notably increasing upon Pso-Mix stimulation (Fig. 6D). Furthermore, immunofluorescence demonstrated co-expression of K17 and AAL-recognized fucosylation in the epidermis of psoriatic skin (Fig. 6E).

Our previous study revealed that upregulation of K17 in psoriasis keratinocytes rely on ubiquitination modification at its K63 site. We hypothesize that FUT11 mediated core fucosylation may regulates K17 stability via ubiquitination modifications. Digestion with PNGase F revealed a distinct band at 25kDa for K17, provides further evidence for glycosylation modification occurring in K17(Fig. 6F). Notably, we observed a reduction in fucosylation of K17 and also a decrease in ubiquitination at its K63 site (Fig. 6G and H). Western blot found that silencing FUT11 significantly decreased the levels of ubiquitylated K17 via K63 linkage in Pso-Mix-treated keratinocytes (Figure S4). Meanwhile, the result of immunoprecipitation assays revealed Pso-Mix treated keratinocytes the binding between Trim21 and K17 was much more robust, whereas treatment with the fucosylation inhibitor (2-FF) and siRNA FUT11 significantly weakened with the binding between Trim21 and K17 (Figure S5). This finding underscores the intrinsic relationship between fucosylation of K17 and its ubiquitination at the K63 site. To further elucidate the impact of FUT11 on the stability of K17, we utilized cycloheximide to assess the degradation dynamics of K17 in keratinocytes stimulated with Pso-Mix. Western blot analysis revealed that FUT11 siRNA significantly shorten the half-life of K17 in keratinocytes cells treated with Pso-Mix (Fig. 6I). This indicates that FUT11 plays a role in diminishing mediating the stability of K17 protein in keratinocytes cells exposed to the Pso-Mix. In summary, our observations suggest that FUT11 may modulate the stability of K17 in keratinocytes cells treated with the Pso-Mix by influencing its ubiquitination modification.

## Discussion

The pathogenesis of psoriasis has increasingly been linked to aberrant post-translational modifications, particularly glycosylation. While previous studies have highlighted the dysregulation of fucosylation in psoriasis [6, 22–25], the mechanistic contributions of this modification remain inadequately explored. In this study, we identified a significant upregulation of fucosylation in keratinocytes derived from psoriatic lesions, which correlated with disease severity. Importantly, pharmacological inhibition of fucosylation in IMQ-induced psoriasis-like mouse model resulted in attenuation of pathological phenotypes, emphasizing the functional significance of this modification. These findings align with emerging evidence that fucosylation plays a crucial role in driving inflammatory and proliferative pathways in psoriasis, positioning it as a critical regulatory node in disease progression.

K17 is specifically overexpressed in psoriatic keratinocytes that amplifies pathogenic signaling [11, 26], serving not merely as a structural protein but also a critical hub in the immune-epidermal crosstalk that drives psoriasis pathogenesis. Its immunoregulatory and antigen-presenting capabilities position K17 as a central pathogenic driver, orchestrating the establishment of a K17/T-cell/cytokine autoimmune positive feedback loop that propagating the pathogenesis of psoriasis [9]. Our previous research revealed that ubiquitination stabilizes K17 in psoriasis, highlighting the importance of post-translational regulation in maintaining its overexpression [12]. However, the role of glycosylation in modulating K17 expression had not been previously investigated. In this study, we provide evidence that K17 undergoes glycosylation, and its expression is tightly coupled to fucosylation. Notably, the depletion of fucosylation significantly reduced K17 protein levels, suggesting a novel regulatory mechanism. We propose that fucosylation serves as a stable modification of K17, promoting its sustained high expression in psoriatic keratinocytes and further mediating the progression of the K17 loop in psoriasis.

This dual-control mechanism may help to explain the persistently high levels of K17 observed in psoriatic epidermis. Mechanistically, we identified FUT11 as the primary fucosyltransferase responsible for K17 modification. Knockdown of FUT11 led to the destabilization of K17, implicating fucosylation as a key factor in maintaining its protein stability. Previous studies have established that fucosylation can modulate the functions of corresponding glycoproteins, such as protein conformation, stability, and functional expression [27–29], which aligns with our observations. Notably, silencing FUT11 diminished the interaction between K17 and Trim21, an E3 ubiquitin ligase previously associated with the ubiquitination of K17. This disruption was accompanied by reduced K17 ubiquitination and accelerated degradation following protein synthesis inhibition with cycloheximide (CHX). These findings suggest a model in which FUT11-mediated fucosylation facilitates Trim21-dependent ubiquitination, stabilizing K17 by diverting it from proteasomal degradation. This dual-control mechanism may help to explain the persistently high levels of K17 observed in psoriatic epidermis. K17 serves as a key mediator linking innate and adaptive immunity, driving the chronic inflammatory process in psoriasis through a self-reinforcing cycle of “expression-immune cell activation-cytokine secretion-K17 upregulation [9, 30, 31]. Given the pivotal role of FUT11 in sustaining the K17/T cell/cytokine autoimmune positive feedback loop, the combinatorial therapeutic strategy involving FUT11-targeted agents and existing biologics holds significant potential to inhibit epidermal-immune system interactions across multiple mechanistic levels.

Emerging evidence has highlighted fucosylation as a therapeutically targetable pathway in both inflammatory and neoplastic diseases. The fucosylation inhibitor 2-FF has been experimentally applied in tumor therapy [32]. Administration of 2-FF as prophylaxis significantly delayed tumor onset and improved overall survival [33]. The application of 2-FF has now been implicated in inflammatory pathologies. While its application in psoriasis remains underexplored. Here, we demonstrated that the administration of 2-FF attenuates psoriasis-like phenotypes both in vitro and in vivo, suggesting broad utility in fucosylation-driven disorders. Notably, targeted silencing of FUT11, the glycosyltransferase responsible for K17 fucosylation, recapitulated the therapeutic effects of 2-FF by suppressing keratinocyte proliferation through downregulation of K17. These findings position 2-FF and FUT11 silencing as a promising candidate for psoriasis therapy.

Our studies delineate a previously unrecognized axis wherein FUT11-mediated α−1,3-fucosylation stabilizes K17 by modulating its ubiquitination. FUT11 depletion disrupts K63-linked ubiquitination of K17. However, there still has limitations that frame critical future directions. First, we linked FUT11 to both fucosylation and K63 ubiquitination of K17, the intermediary factors facilitating this crosstalk—such as adaptor proteins or regulatory enzymes—remain unidentified. Second, the in vivo role of FUT11 in psoriasis pathogenesis requires validation using keratinocyte-specific FUT11 knockout models to exclude compensatory effects from other fucosyltransferases. Finally, Aleuria Aurantia Lectin (AAL) exhibits broad specificity, capable of simultaneously binding to both α1,3- and α1,6-fucose linkages. Although the stability expression of FUT8 (an enzyme mediating core fucosylation) and the specific effects of FUT11-targeted intervention in our study collectively rule out the dominant role of core fucosylation (FUT8-mediated) in AAL signaling, definitive validation of FUT11 as a direct target still requires identification of K17 glycosylation sites via α1,3-fucose-specific antibodies or mass spectrometry. This approach will conclusively main attribute AAL signals to FUT11 activity, thereby excluding the contribution of α1,6-fucose linkages.

## Conclusion

In conclusion, we identify FUT11 as a critical driver of K17 stabilization through both fucosylation and ubiquitination modifications, offering a mechanistic explanation for the pathogenic overexpression of K17 in psoriasis. Our findings not only expand the understanding of post-translational crosstalk in disease but also nominate FUT11 and its downstream effector K17 as actionable therapeutic targets. The efficacy of 2-FF in preclinical models further validates fucosylation inhibition as a viable therapeutic strategy, urging translational efforts to refine this approach for clinical use.

## Supplementary Information

Supplementary Material 1.

Supplementary Material 2.

Supplementary Material 3.

## Data Availability

No datasets were generated or analysed during the current study.
