# Supplementary material for: FUT11-Driven fucosylation coordinates K63 ubiquitination of keratin 17 to sustain psoriatic keratinocytes hyperproliferation
Source: Cell Commun Signal. 2025 Oct 22;23:456. doi: 10.1186/s12964-025-02422-6 (PMC12542314; doi:10.1186/s12964-025-02422-6)
Supplement: Supplementary file 1 — Supplementary Material 1. [file 12964_2025_2422_MOESM1_ESM.pdf]

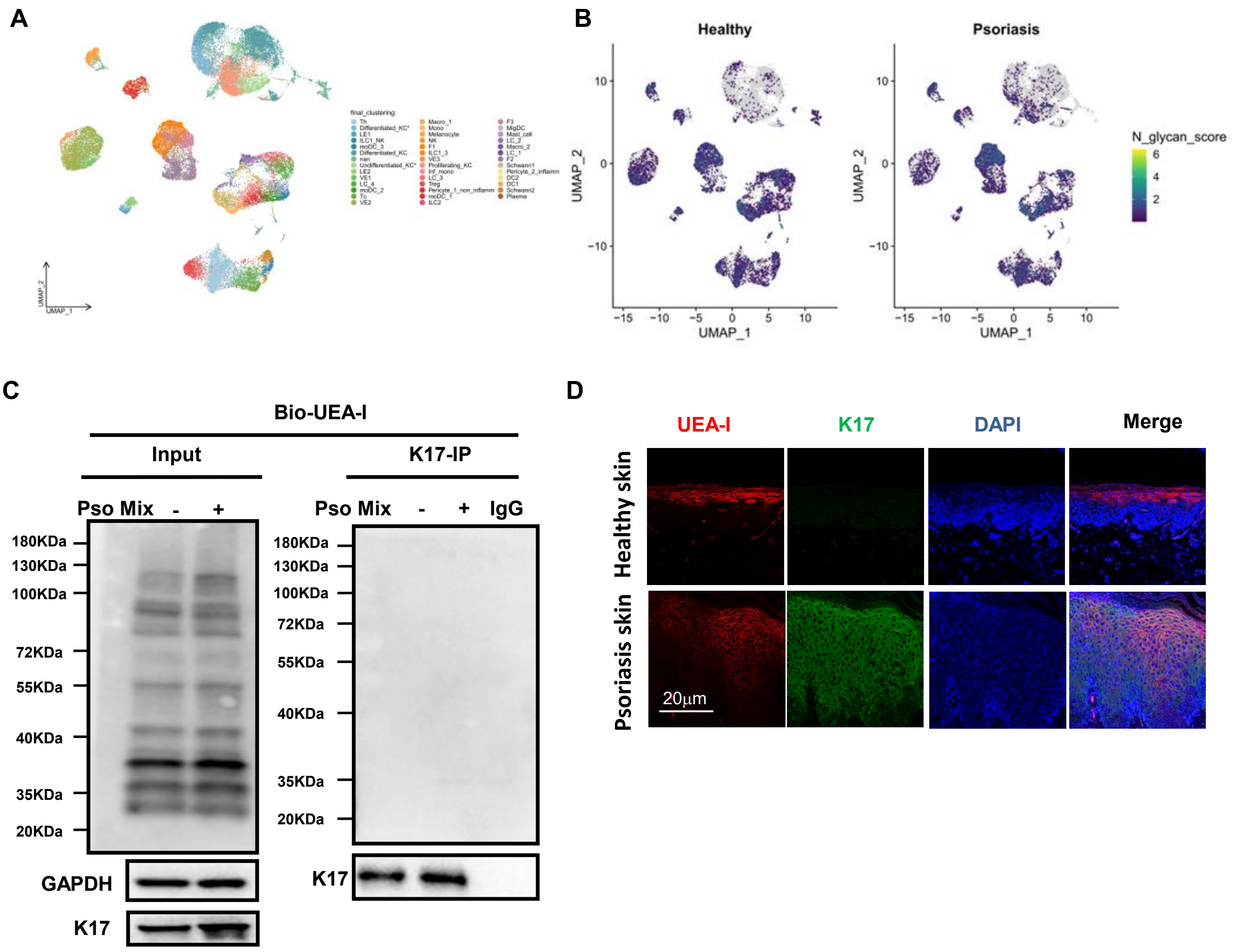

Figure S1 | The psoriatic keratinocytes (KCs) is not occurred UEA-I binding Fucalpha(1,2) containing oligosaccharides. (A)Uniform Manifold Approximation and Projection (UMAP) visualization showing 50000 single cells from 5 healthy donors and 3 psoriatic patients scRNA-seq data sets. According to the identified cell marker, 44 clusters of skin were identified. Dots of different colors represent cells in corresponding clusters.(B) All single-cell N-glycan biosynthesis activity was scored using the AddModuleScore function in Seurat. UMAP plots visualize cell score distribution with color gradients reflecting scoring intensity, showcasing the spatial distribution of cell scores. (C) Fucosylation modification of K17. Cells were immunoprecipitated with anti-K17 and immunoblotted as shown. (D) Immunofluorescence staining for AAL and K17, AAL(Red),K17(green),Scale bar = 20 μm.

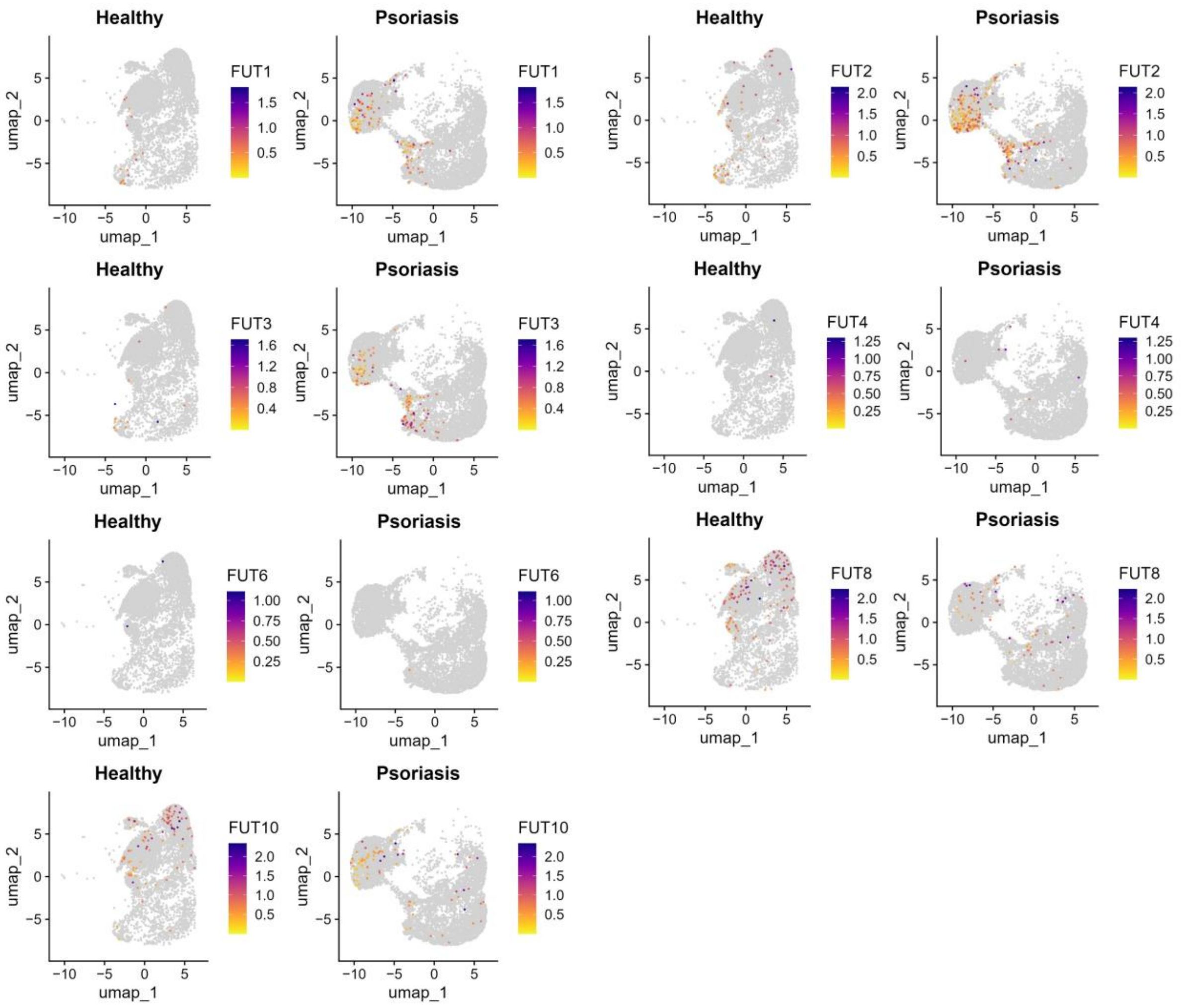

Figure S2 | UMAP plot visualizes the expression levels of fucosyltransferase genes (FUT1， FUT2,FUT3,FUT4,FUT6,FUT8 and FUT10)across keratinocyte subpopulations, with FUT5 and FUT9 exhibiting negligible expression. Gene expression intensity is represented through a color gradient, where darker colors correspond to higher expression levels.

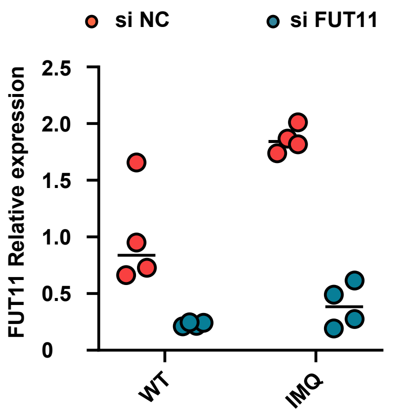

Figure S3 | Relative mRNA expression of FUT11 in IMQ-induced psoriasis-like mice ears. Results were shown as mean ± SD. *P < 0.05, *P < 0.01, ***P < 0.001.

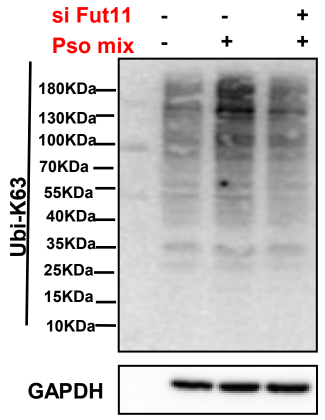

Figure S4 | Western blot analysis the levels of ubiquitylated via K63 linkage by silencing FUT11.

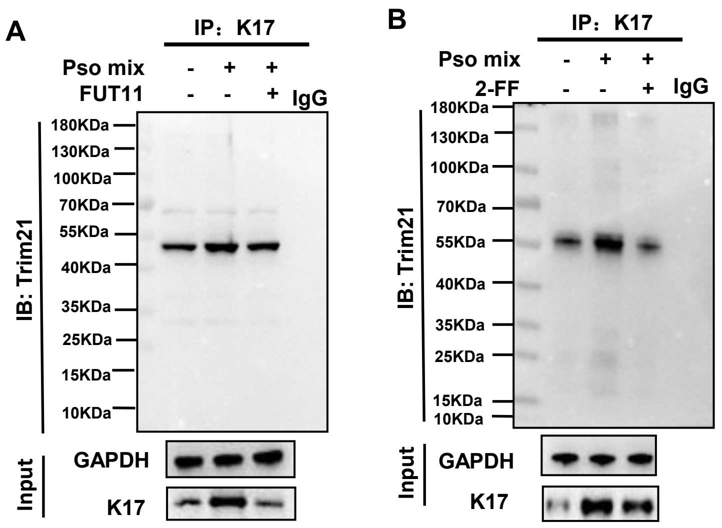

Figure S5 | Immunoprecipitation assays with anti-K17 antibody for Trim21 in keratinocytes treated with fucosylation inhibitor (2-FF) and siRNA FUT11.
