# Supplementary figures and images for: FUT11-Driven fucosylation coordinates K63 ubiquitination of keratin 17 to sustain psoriatic keratinocytes hyperproliferation
Source: Cell Commun Signal. 2025 Oct 22;23:456. doi: 10.1186/s12964-025-02422-6 (PMC12542314; doi:10.1186/s12964-025-02422-6)

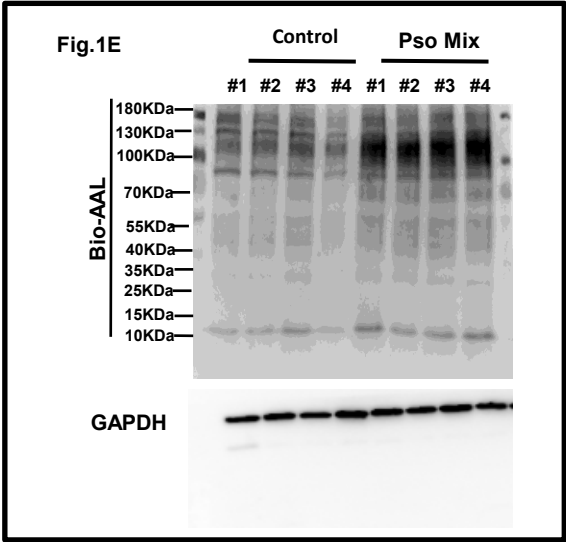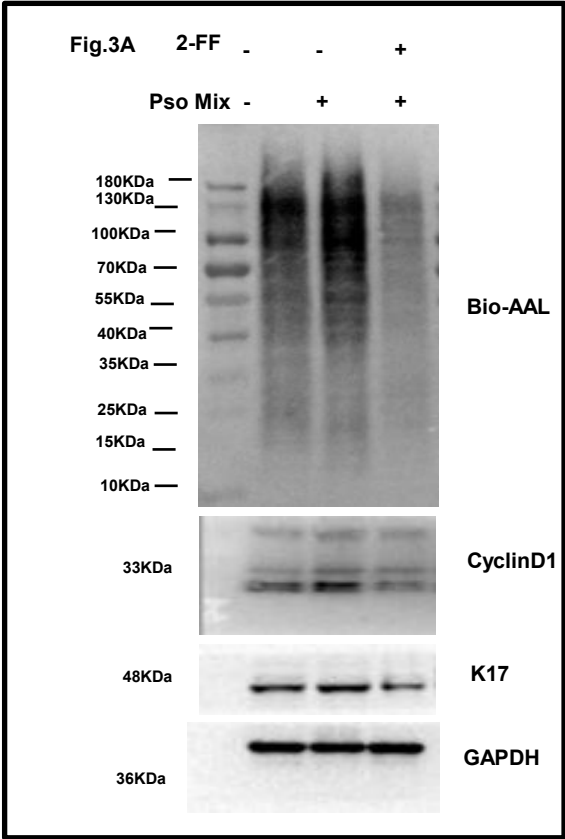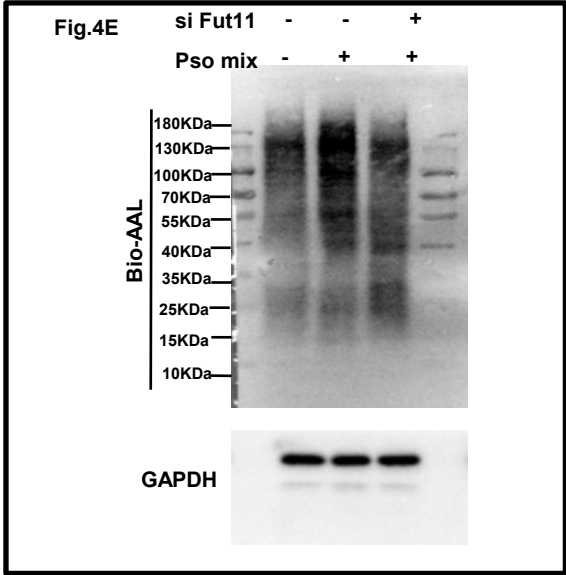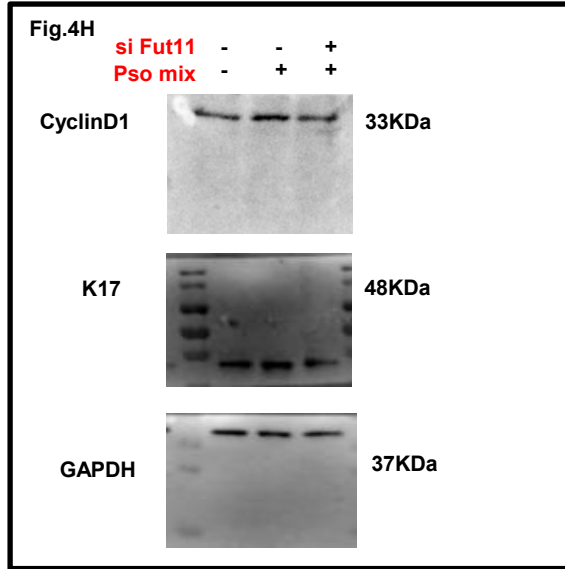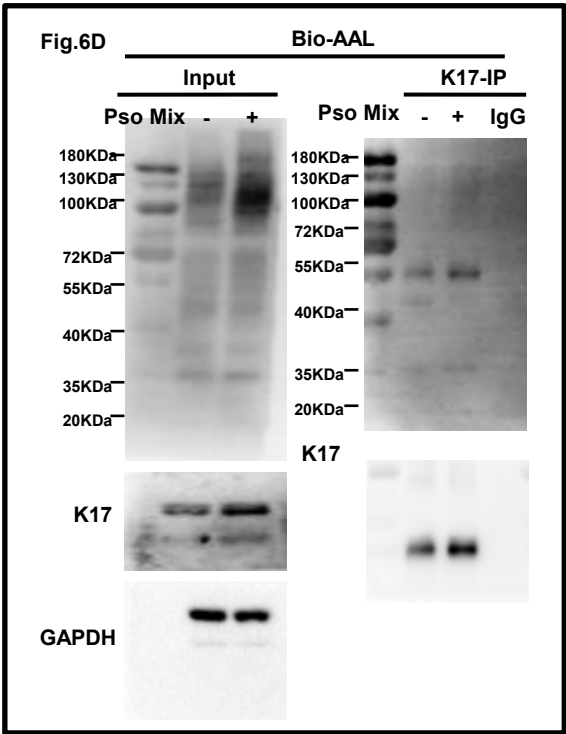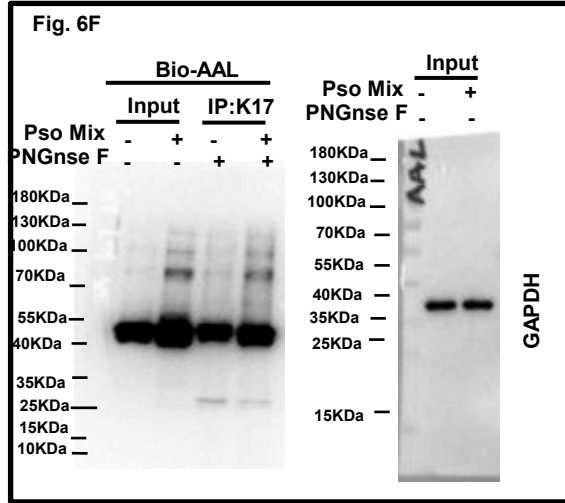

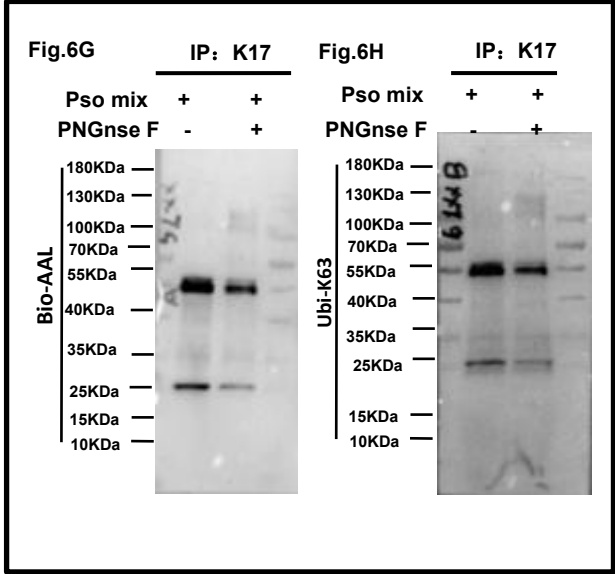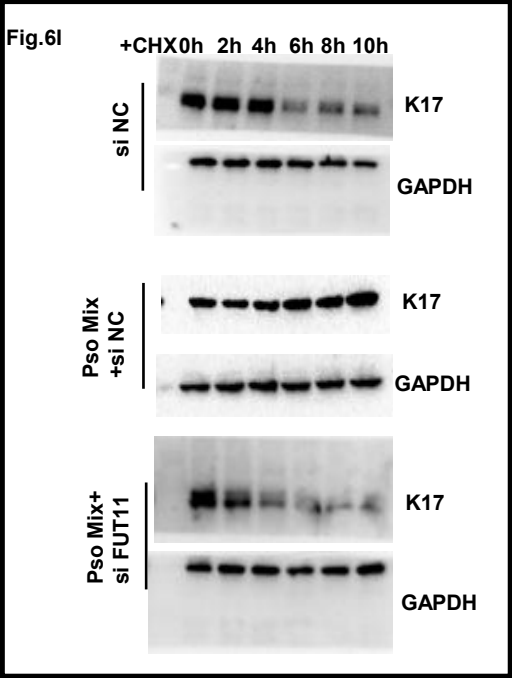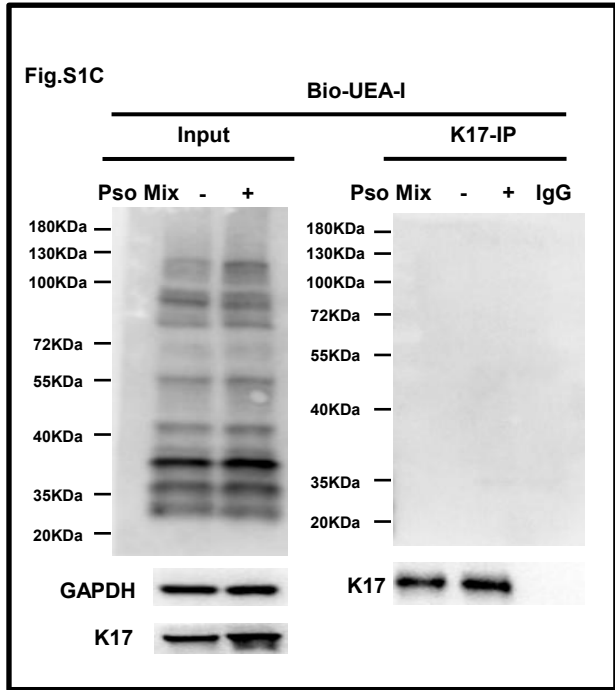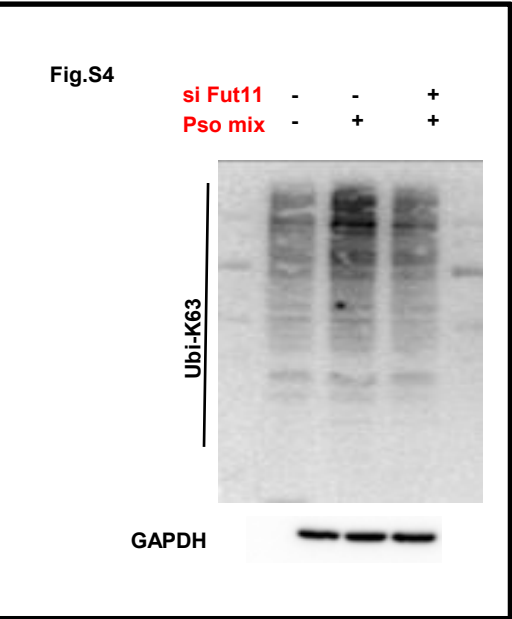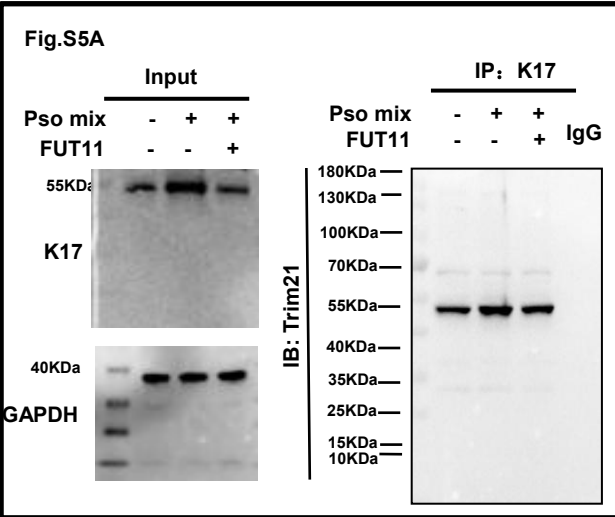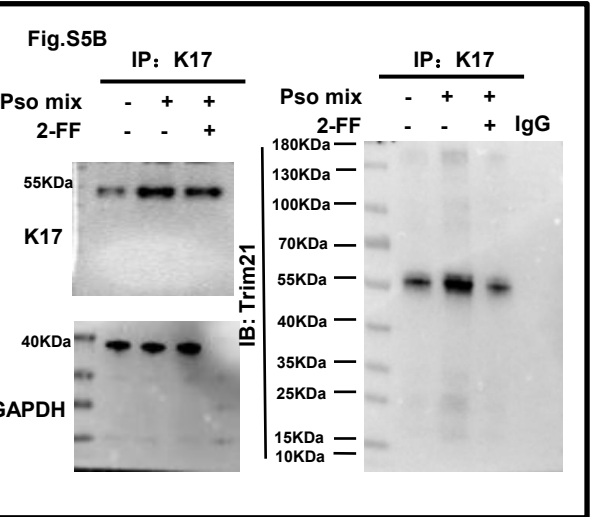

Supplement: Supplementary file 2 — Supplementary Material 2. [file 12964_2025_2422_MOESM2_ESM.pdf]
