# Supplementary material for: FUT11-Driven fucosylation coordinates K63 ubiquitination of keratin 17 to sustain psoriatic keratinocytes hyperproliferation
Source: Cell Commun Signal. 2025 Oct 22;23:456. doi: 10.1186/s12964-025-02422-6 (PMC12542314; doi:10.1186/s12964-025-02422-6)
Supplement: Supplementary file 3 — Supplementary Material 3. [file 12964_2025_2422_MOESM3_ESM.docx]

**Table S1 Primer information for qPCR**

|  | **Primer pairs (5’-3’)** |
| --- | --- |
| Human GAPDH | AGAAGGCTGGGGCTCATTTG |
|  | AGGGGCCATCCACAGTCTTC |
| Human K17 | GAGCAGCAGAACCAGGAATACA |
|  | TTTCTTGTACTGAGTCAGGTGGGC |
| Human cyclinD1 | GATCAAGTGTGACCCGGACT |
|  | CTTGGGGTCCATGTTCTGCT |
| Human PCNA | CGGTTACTGAGGGCGAGAAG |
|  | CGACCGGCTGAGACTTGC |
| Human FUT1 | AGCAACGGCATGGAGTGGTGTA |
|  | AAGCCGAAGGTGCCAATGGTCA |
| Human FUT2 | CTACCACCTGAACGACTGGATG |
|  | AGGGTGAACTCCTGGAGGATCT |
| Human FUT3 | CCGCACUGCUAUUUCAGCUGCUGGU |
|  | ACCAGCAGCUGAAAUAGCAGUGCGG |
| Human FUT4 | GGGTTTGGATGAACTTCGAGTCG |
|  | GGTAGCCATAAGGCACAAAGACG |
| Human FUT6 | GCUGUCUGACCACGCUGCUGUUUCA |
|  | UGAAACAGCAGCGUGGUCAGACAGC |
| Human FUT8 | GACAGAACTGGTTCAGCGGAGA |
|  | GCAGTAGACCACATGATGGAGC |
| Human FUT10 | CTAACCAGCGACTTCTGACAGC |
|  | CCCATCTTTTGGGTGGTAAGCC |
| Human FUT11 | ACACCTGGCTTTGGCAATGTGG |
|  | GTGGATCATGGCAGTGAGAGCT |
| Human SLC35C1 | TGCTCAAGCAGACCACCTCCTT |
|  | AGATGGCGTTGAGCGAGACACA |
| Human GMDS | TGAGTTCCTGCTGGAGAAAGGC |
|  | CAAGGCAGGTACTGTCAGTGAG |
| Human F10 | TGGTGGAACCATTCTGAGCGAG |
|  | CGGTTGTGCTTGATGACCACCT |
| Human TSTA3 | AAGCCATCCAGAAGGTGGTAGC |
|  | GGTTGGACCTTCTCAAACAGGG |
| Mouse GAPDH | GGTGAAGGTCGGTGTGAACG |
|  | CTCGCTCCTGGAAGATGGTG |
| Mouse S100A9 | ATACTCTAGGAAGGAAGGACACC |
|  | TCCATGATGTCATTTATGAGGGC |
| Mouse K17 | ACCACCATCCGCCAGTTTAC |
|  | CTCCCAAGAGCACTACCCAG |
| Mouse IL-17 | AATCCTGGTCCTTCGGAGGG |
|  | GCCAACTTTTAGGAGCATCTTCT |
| Mouse PCNA | ACCTCACCAGCATGTCCAAAA |
|  | GCTGTACTCCTGTTCTGGGA |
| Mouse CCL20 | CAGGCAGAAGCAAGCAACTAC |
|  | AGCTTCATCGGCCATCTGTC |

**Table S2. SiRNAsequences**

| **Gene symbol** |  | **siRNASequences** |
| --- | --- | --- |
| Human sense FUT11siRNA | Sense | GUGGAAGAGAUUCCUGAGAAUdTdT |
|  | Anti-Sense | AUUCUCAGGAAUCUCUUCCACdTdT |
| Control sense siRNA | Sense | UUCUCCGAACGUGUCACGUUUC |
|  | Anti-Sense | GAAACGUGACACGUUCGGAGAA |
| Mouse sense FUT11 siRNA | Sense | GGACUGGAUGCCCAAUAAUCAdTdT |
|  | Anti-Sense | UGAUUAUUGGGCAUCCAGUCCdTdT |
| Control sense siRNA | Sense | UUCUCCGAACGUGUCACGUTT |
|  | Anti-Sense | ACGUGACACGUUCGGAGAATT |
